# Combined surgical and medical management of a broad ligament ectopic pregnancy: A case report

**DOI:** 10.1016/j.crwh.2021.e00316

**Published:** 2021-04-19

**Authors:** Amanda J. Compadre, Erinma P. Ukoha, Wenjia Zhang

**Affiliations:** Department of Obstetrics, Gynecology, & Reproductive Sciences, University of California San Francisco, USA

**Keywords:** Ectopic, Pregnancy, Laparoscopy

## Abstract

Broad ligament ectopic pregnancies rare. Several case reports illustrate treatment with laparoscopy. A 31-year-old woman, gravida 2 para 1, presented at 6 weeks 6 days of gestation with vaginal bleeding. She had a β-hCG level of 7424 IU/L and ultrasound showed a 1.8 cm ectopic pregnancy with fetal cardiac activity in the left adnexa. Diagnostic laparoscopy revealed a left broad ligament ectopic pregnancy. The products of conception were removed surgically, and she received prophylactic two-dose methotrexate. Her β-hCG returned to non-pregnant levels within one month. This is the first case report to describe laparoscopy with postoperative prophylactic methotrexate for successful treatment of a broad ligament pregnancy. This treatment protocol with methotrexate can be considered in future cases.

## Highlights

• Broad ligament ectopic pregnancies are rare and often diagnosed intraoperatively.
• Several case reports document successful treatment of these pregnancies with surgery.
• Prophylactic methotrexate can be considered if there is concern for retained trophoblastic tissue.

## 1 Introduction

Although 90% of ectopic pregnancies are located in the fallopian tube, they can also occur in the cervix, ovary, interstitially, in a Cesarean section scar, intramurally, or abdominally [1]. Abdominal pregnancies, in which implantation occurs in the peritoneal cavity outside the uterine cavity or fallopian tubes, comprise only 1% of ectopic pregnancies, but are associated with a high degree of morbidity and mortality [2]. A review of abdominal pregnancies revealed a maternal mortality rate of 5.1 per 1000 pregnancies, which is 7.7 times greater than the mortality rate of tubal ectopic pregnancies and 90 times higher than that of intrauterine pregnancies [2]. Locations of abdominal ectopic pregnancies include pouches around the uterus, abdominal organs, omentum, spleen, retroperitoneal spaces, and abdominal wall.

An abdominal ectopic pregnancy located at the broad ligament (within the two layers) is rare [3]. It occurs in about 1 in 300 total ectopic pregnancies and can be difficult to diagnose on imaging due to the proximity of the broad ligament to the fallopian tube [4,5]. Often the diagnosis made by direct visualization at the time of surgery [5]. Given its rarity, there is limited literature on the management of broad ligament ectopic pregnancies. Most case reports describe patients who underwent laparotomy; however, there is an increasing number of cases treated laparoscopically [5]. There are no known cases of broad ligament pregnancies treated medically with methotrexate or potassium chloride alone. Here, we present the case of a patient diagnosed with a broad ligament ectopic pregnancy during laparoscopy who was successfully managed with a novel approach of surgical removal and prophylactic methotrexate.

## 2 Case Presentation

A 31-year-old woman, gravida 2 para 1, with unremarkable medical, surgical and family history, presented to clinic at 6 weeks 6/7 days of gestation (based on a sure, regular last menstrual period) with a one-week history of light vaginal bleeding. In clinic, her urine pregnancy test was positive. A bedside transabdominal ultrasound showed no intrauterine pregnancy and no adnexal masses. Her serum β-hCG was tested and reported the next day at 7424 IU/L. She was referred to the emergency department for further evaluation. She denied abdominal pain, fever, chills, or lightheadedness and was hemodynamically stable. On exam, her abdomen was soft, nondistended, nontender without rebound or guarding. Pelvic exam was notable for scant bloody discharge seen at the cervical os; no adnexal tenderness or fullness was appreciated. Laboratory examination revealed a serum β-hCG level of 10,931 IU/L, normal blood counts, with a hemoglobin of 12.9 g/dL and hematocrit of 38.8%, normal metabolic panel, and Rh-positive status.

**Fig. 1:**
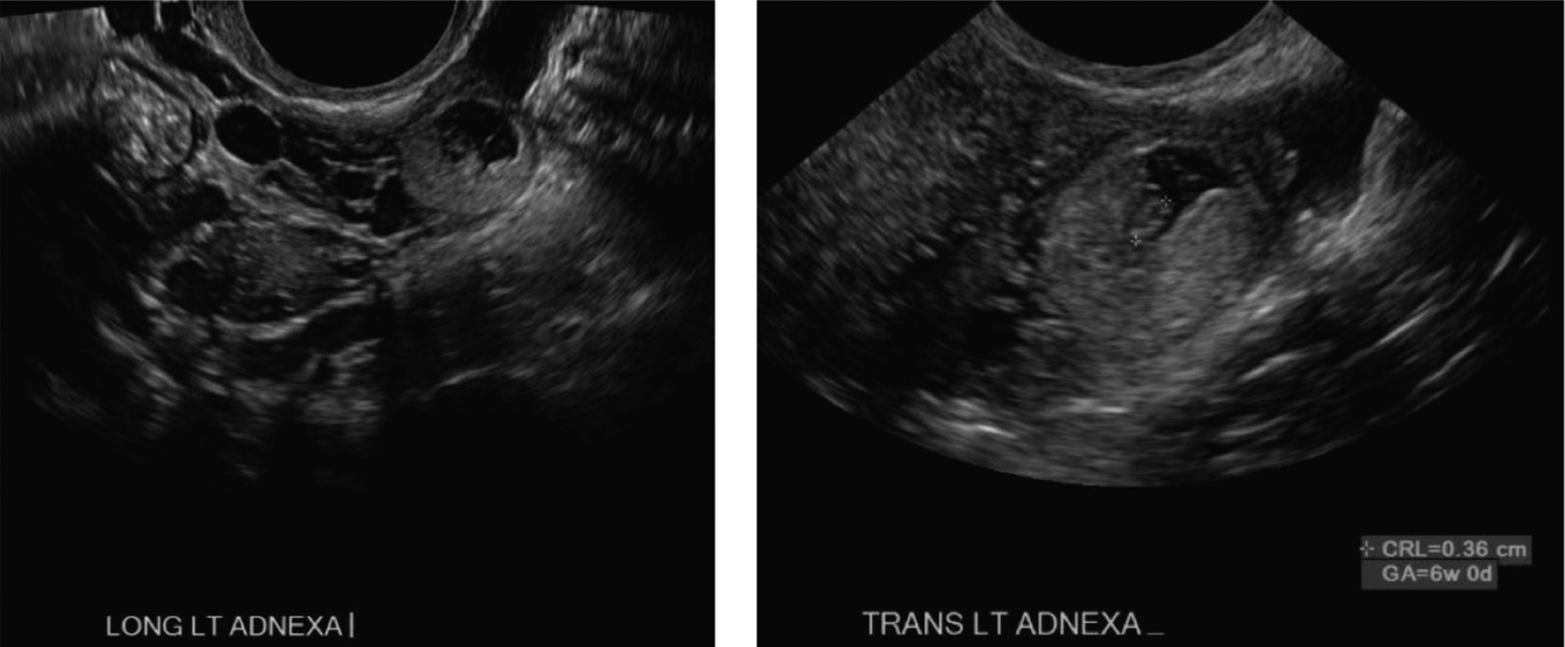
A. Transvaginal ultrasonography of left adnexal ectopic pregnancy demonstrating left ovary separate from ectopic pregnancy. B. Left adnexal ectopic pregnancy with crown rump length at 6 weeks 0 days.

**Fig. 2:**
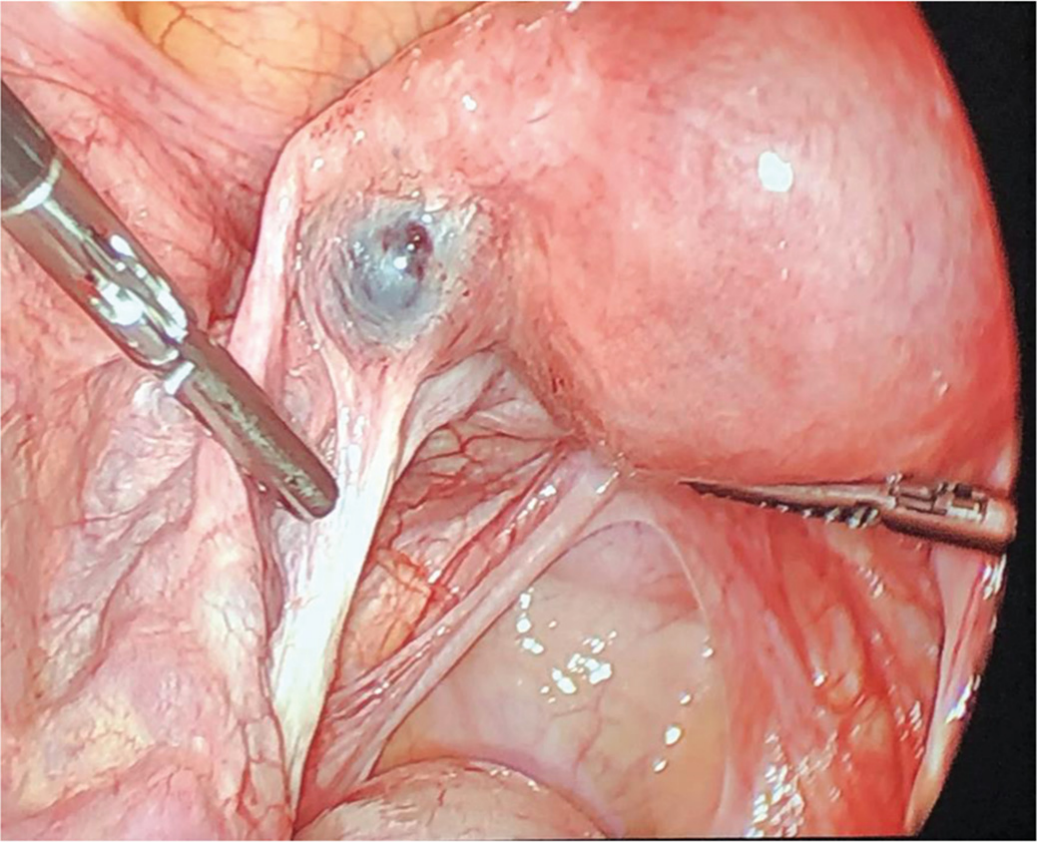
Intraoperative image of left broad ligament ectopic pregnancy between the left fallopian tube and utero-ovarian ligament, unruptured and without overlying myometrium.

**Fig. 3:**
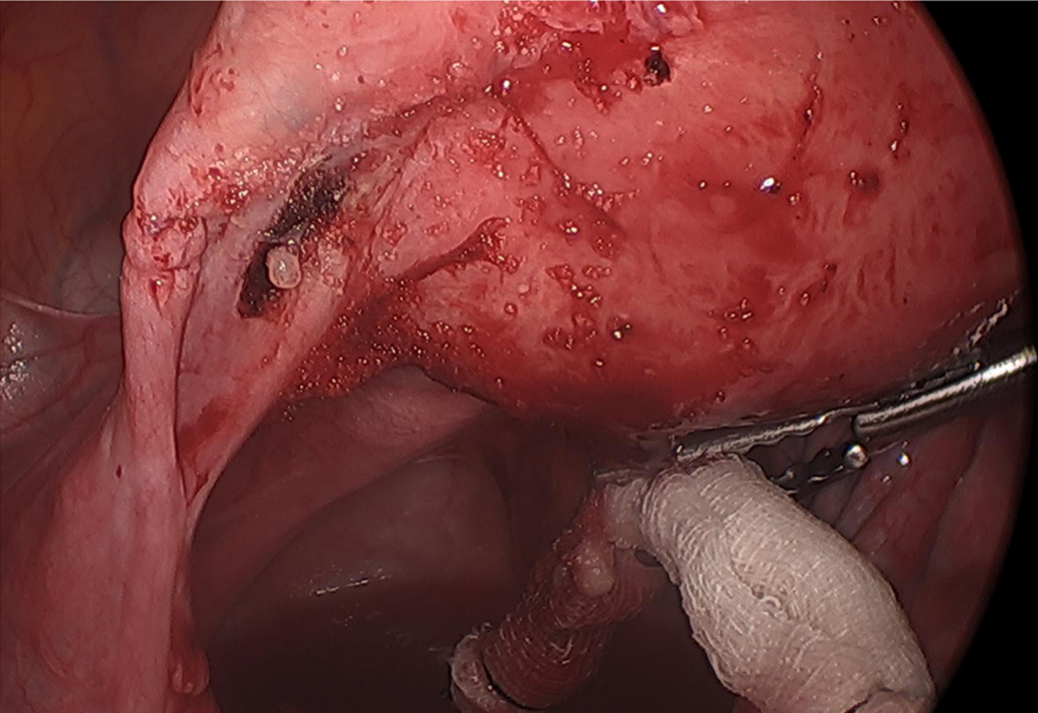
Intraoperative image after surgical removal of left broad ligament ectopic pregnancy.

Formal transvaginal ultrasound showed no intrauterine fluid collection and within the left adnexa a 1.8 × 1.5 × 1.5 cm round structure with eccentric thick echogenic soft tissue likely representing chorionic tissue. There was demonstration of a yolk sac and fetal pole with cardiac activity at 125 beats per minute. Bilateral ovaries were normal in appearance [Fig. 1]. There was trace free fluid within the pelvic cul-de-sac. Given her elevated β-hCG and ultrasound findings consistent with unruptured left tubal ectopic pregnancy with fetal cardiac activity, she was recommended and consented for a diagnostic laparoscopy with unilateral salpingectomy according to findings. Laparoscopy was undertaken in the usual fashion. Intraoperative findings revealed a 2 cm ectopic pregnancy in the left broad ligament, lateral to the uterus occupying the space between the left fallopian tube and utero-ovarian ligament, unruptured and without overlying myometrium [Fig. 2]. There was minimal blood in the cul-de-sac. Otherwise, the uterus and bilateral tubes and ovaries appeared normal. An incision was made overlying the gestational sac using the monopolar hook. The broad ligament was not explored. The products of conception were removed in fragments using Maryland forceps, and the area was copiously irrigated. The broad ligament was rendered hemostatic using coagulation and Floseal. Due to oozing along the tissue edges, the area was reapproximated with a figure-of-eight stitch using 2–0 vicryl [Fig. 3]. The estimated blood loss was 150 mL. The patient tolerated the procedure well and was discharged on postoperative day 1. Final pathology showed immature placental villi.

Since the ectopic pregnancy was in the broad ligament and it was unclear if all products of conception had been removed, methotrexate was recommended to ensure complete resolution of the pregnancy. She received a two-dose regimen of intramuscular methotrexate (50 mg/m2 each). She received her first injection on the day of the surgery prior to discharge and the second dose on day 4. Her β-hCG levels on days 1, 4, and 7 were 6948 IU/L, 1718 IU/L, and 1348 IU/L, respectively. Given the adequate decrease in her β-hCG level of greater than 15%, her β-hCG levels were trended on a weekly basis. Her serum β-hCG returned to non-pregnant levels over the following month, and she was doing well at her eight-week follow-up visit.

## 3 Discussion

A broad ligament ectopic pregnancy is a rare type of pregnancy that is difficult to diagnose on imaging and can result in significant morbidity due to rupture and hemoperitoneum [6]. The misdiagnosis of broad ligament ectopic pregnancies can lead to later presentations, in the second and third trimesters, and can even result in live births [3]. However, with improvement in diagnostic tools such as serum β-hCG and transvaginal sonography, ectopic pregnancies are diagnosed at much earlier gestational ages. Despite these advancements, broad ligament pregnancies are usually not diagnosed until surgery due to the lack of specific characteristics on imaging. In a recent case review of 17 broad ligament pregnancies, all but one was diagnosed intraoperatively [3]. The risk factors for broad ligament pregnancies are similar to those for tubal ectopic pregnancies and include tubal abnormalities, previous ectopic pregnancy, previous salpingectomy, pelvic infection, endometriosis, adhesions, assisted reproductive technology (ART), and presence of intrauterine devices [3]. Although the incidence of broad ligament ectopic pregnancies is low, due to increasing use of ART it is likely it will rise.

In our review of the literature, we identified 19 case reports of broad ligament ectopic pregnancies after 1990. Of these, 8 were managed laparoscopically, and the remainder were managed with laparotomy [[3], [4], [5], [6]]. Our case is the ninth documented case of a broad ligament ectopic managed laparoscopically [Table 1] and is the first to use prophylactic methotrexate postoperatively.

**Table 1:** Summary of case reports of broad ligament ectopic pregnancies managed laparoscopically.

Case	Study	Age (years)	Parity	Risk Factors	Presentation	Gestational Age (weeks)	β-hCG (international units/L)	Ultrasound Findings	Treatment and Findings	Follow up
1	Olsen (1997) [11]	26	G3P1011	None	Vaginal spotting	7	22,140	Right adnexal mass consistent with right tubal ectopic pregnancy	Laparoscopy with <1 cm intraligamentary pregnancy, managed with bipolar scissors excision and electrocautery	β-hCG followed serially, 10 months later had miscarriage
2	Siow (2004) [12]	25	Unknown	None	Asymptomatic	10	55.8	5.5 cm right adnexal mass with crown rump length (CRL) measuring 8 weeks, no fetal cardiac motion	Laparoscopy with 6.5 cm broad ligament pregnancy, managed with vasopressin injection and excision	Doing well at four week follow up
3	Apantaku (2006) [13]	33	G4P1021	Previous laparotomy and bilateral salpingectomy, IVF pregnancy	Not mentioned	8	NA	Right adnexal mass with CRL of 3 mm suggestive of ectopic pregnancy	Laparoscopy with 2 cm broad ligament pregnancy below remnant of right tube, excised with surgiswip sutures and monopolar energy	Doing well at six week follow up
4	Cormio (2006) [14]	28	G0	Ipsilateral salpingo-ophorectomy	Abdominal pain and vaginal bleeding	10	35,760	5 cm round mass over left adnexal region	Laparoscopy with broad ligament pregnancy removed en bloc with myometrium	None
5	Cheung CS (2014) [3]	30	G4P0030	Ipsilateral salpingectomy	Vaginal bleeding	6	5665	2 cm right mass concerning for pregnancy at stump of fallopian tube, CRL measuring 5w5d with slow fetal pulse	Laparoscopy with 3 cm broad ligament pregnancy excised and suture to obliterate space	β-hCG returned to non-pregnant levels at four weeks
6	Nayar (2016) [5]	25	G0	None	Vaginal spotting and abdominal pain	6	24,719	3.5 cm right adnexal mass with fetal pole measuring 7 weeks and cardiac activity	Laparoscopy with 3.5 cm broad ligament pregnancy excised and right salpingectomy	Lost to follow up after six-week post op checkup
7	Sassi (2018) [4]	32	G2P1	None	Asymptomatic	7	26,784	Ectopic gestational sac and live extrauterine embryo with CRL of 12 mm	Laparoscopy with gestational sac in broad ligament excised	Followed for 28 days post operatively and β-hCG levels <15
8	Cosentino (2017) [6]	35	G3P1	None	Asymptomatic	12	Not Stated	Ectopic abdominal pregnancy with a live fetus in the left parauterine side	Laparoscopy with excision of broad ligament pregnancy	None
9	Our case (2020)	31	G2P1	None	Vaginal bleeding	6	7424	1.8 cm left adnexal mass with yolk sac and fetal pole and CRL of 36 mm and cardiac activity	Laparoscopy with 2 cm broad ligament pregnancy excised and prophylactic methotrexate	β-hCG returned to non-pregnant levels after one month, doing well at eight week follow up

As in the majority of other cases, our patient presented with vaginal bleeding. She was hemodynamically stable, with a β-hCG level of over 7000 IU/L, and with ultrasound evidence of a 2 cm left tubal ectopic pregnancy. Because she had several relative contraindications to medical management of an ectopic pregnancy, we proceeded with a diagnostic laparoscopy [7]. Similar to the majority of case reports, we did not make the diagnosis of a broad ligament pregnancy until direct visualization intraoperatively. We decided to surgically excise the pregnancy tissue, which was subsequently removed in fragments. Given the proximity to the utero-ovarian artery and fallopian tube, we did not attempt to remove the pregnancy en bloc due to concern for bleeding and potential damage to the fallopian tube.

The decision for prophylactic methotrexate was driven by the concern for retained trophoblastic tissue between the leaves of the broad ligament. Retained products of conception have been associated with subsequent ectopic rupture, need for reoperation, and persistently elevated β-hCG levels [8]. Previous studies regarding the use of methotrexate prophylactically after salpingostomy for tubal ectopic pregnancies have shown that the incidence of persistent ectopic pregnancy was significantly reduced after methotrexate – relative risk 0.13 (95% CI 0.02, 0.97) [8]. A decision analysis published in 2001 comparing observation against prophylactic methotrexate for women after salpingostomy for treatment of tubal ectopic showed that prophylactic methotrexate results in fewer cases of tubal rupture (0.4% vs 3.7%), fewer procedures (1.9% vs. 4.7%), and is more cost efficient compared with observation alone [9]. Although our case did not involve a tubal ectopic, we believed it was reasonable to offer the patient prophylactic methotrexate given the limited literature on broad ligament ectopic pregnancies and the significant morbidity associated with rupture. We used the two-dose protocol as a meta-analysis in 2017 had reported higher treatment success overall (as defined by the individual studies) with this regimen compared with the single-dose protocol – odds ratio 1.85 (95% CI 1.13, 3.00) [10]. Our patient tolerated the two-dose regimen without complications and had a reassuring decline in β-hCG over the next month. Of note, it is unclear if there would have been the same regression of β-hCG levels without methotrexate. However, given that the patient had β-hCG levels in the thousands postoperatively, they may have taken significantly longer to return to non-pregnant levels without additional treatment.

In summary, there is little data to guide management of broad ligament pregnancies, but several case reports illustrate safe and successful treatment with laparoscopic removal. Our case is the first to describe laparoscopic removal followed by prophylactic two-dose methotrexate. Future cases of broad ligament ectopic pregnancies can consider utilizing prophylactic methotrexate postoperatively as treatment of these rare ectopic pregnancies.
